# The High Rates of Comorbidity among Neurodevelopmental Disorders: Reconsidering the Clinical Utility of Distinct Diagnostic Categories

**DOI:** 10.3390/jpm14030300

**Published:** 2024-03-11

**Authors:** Eleni Bonti, Irini K. Zerva, Christiana Koundourou, Maria Sofologi

**Affiliations:** 1 First Psychiatric Clinic, School of Medicine, Faculty of Health Sciences, Aristotle University of Thessaloniki, “Papageorgiou” General Hospital, Pavlos Melas, 564 29 Agios Pavlos, Greece; bonti@auth.gr; 2 School of Education, Special Education Department, University of Nicosia, Nicosia 2417, Cyprus; 3 First Psychiatric Clinic, School of Medicine, Aristotle University of Thessaloniki, 541 24 Thessaloniki, Greece; 4 Psychology Department, School of Health Sciences, Neapolis University Pafos, Paphos 8042, Cyprus; c.koundourou@nup.ac.cy (C.K.); m.sofologi@nup.ac.cy (M.S.); 5 Department of Early Childhood Education, Education School, University of Ioannina, 451 10 Ioannina, Greece; 6 Institute of Humanities and Social Sciences, University Research Center of Ioannina (URCI), 451 10 Ioannina, Greece

**Keywords:** neurodevelopmental disorders, comorbidity, distinct diagnostic categories, *DSM-5*

## Abstract

The boundaries between neurodevelopmental disorders are often indistinct, even among specialists. But do these boundaries exist, or do experts struggle to distinguish and categorize symptoms in order to arrive at a dominant diagnosis while comorbidity continually leaves questions about where each disorder ends and begins? What should be reconsidered? The introduction of the term ‘spectrum of neurodevelopmental disorders’ could pave the way for a re-appraisal of the clinical continuum of neurodevelopmental disorders. This study aims to highlight the problems that emerge in the field of the differential diagnosis of neurodevelopmental disorders and propose a renegotiation of the distinctiveness criteria.

## 1. Introduction

Looking at the history of the diagnostic classifications of what are, today, known as ‘Neurodevelopmental Disorders’ (NDDs) in the *DSM-5* [1], it becomes obvious that classifying each of these disorders into a clear-cut, distinct diagnostic category has not been an ‘easy task’.

In addition, there are extremely high rates of comorbidity among the distinct disorders of this diagnostic grouping (i.e., NDDs), as well as between NDDs and other psychiatric disorders (e.g., Emotional and or Behavioral Disorders—EBDs). Moreover, there is a consistent phenotypic overlap of typical characteristics evident in two or more of these disorders (e.g., Specific Learning Disorder (SLD), Attention Deficit Hyperactivity Disorder (ADHD), and Communication Disorders (CD)). These issues cause enormous frustration to clinicians, indicating that both the criteria of the different diagnostic categories as discrete entities and, in some cases, the diagnostic categories themselves might not be as clear-cut as has been argued.

The possibility that there might be a ‘core’ (cognitive, genetic, neurological, or other) deficit in NDDs, along with the recent research conceptualizations, which hypothesize that there might be a common genetic cause of NDDs (i.e., specific genes and/or variants might be responsible for NDDs), complicates the overall ‘picture’ even more. Other theoretical assumptions implying a genetic neurodevelopmental continuum in the presence of NDDs and/or several perspectives that involve environmental factors that might be responsible for the incurrence of NDDs add further considerations to this issue.

On the other hand, using discrete diagnostic categories (mainly based on behavioral phenotypes) provides clinicians with a relatively ‘clear’ context for defining children’s problems or deficits in several developmental domains, communicating these deficits to parents and educators, and planning appropriate treatments and therapeutic/intervention programs.

Nonetheless, placing a child in a specific diagnostic category runs the risk of failing to capture other important aspects of the individual’s overall deficient or over-efficient developmental areas of functioning. An example of such a risk or a diagnostic pitfall is the case of ‘twice-exceptional’ learners, i.e., learners with a dual diagnosis of giftedness and an NDD.

All the above considerations highlight the need for further future research that would aim to better define the exact nature and prevalence of NDDs, address and closely examine the possible causes of high rates of comorbidity among NDDs and develop a more comprehensive diagnostic and categorization system. Such a system would allow more appropriate interventions to be planned to provide these individuals with better educational environments that will meet their unique needs but, at the same time, would allow them to express their full potential and improve their quality of life.

## 2. The Diagnostic Grouping of NDDs in *DSM-5*

In the revised version of the *DSM-5* [1], the diagnostic grouping of ‘Neurodevelopmental Disorders’ (NDDs) comprises the following distinct disorders: Intellectual Disability (ID), Communication Disorders (CD), Autism Spectrum Disorder (ASD), Attention Deficit Hyperactivity Disorder (ADHD), Specific Learning Disorders (SLD), and Developmental Motor Disorders (including tic disorders).

NDDs constitute a group of clinical symptoms and conditions with onset in the developmental period that usually emerge by the age of five years in 5–9% of children and accompany the individual for the rest of their life. They involve deficits that might impair various areas or domains of the individual’s development and functioning and the person’s overall quality of life since they negatively affect almost every aspect of their development and functionality on a personal, academic, social, and professional level [2].

Hence, most NDD disorders of childhood development are usually not limited to a single diagnosis but often cause additional difficulties during the next stages of the individual’s life (i.e., adolescence and adulthood), such as social, emotional, or behavioral problems [3].

According to recent research, the varied interpersonal characteristics, emerging comorbidity issues, and different developmental backgrounds of individuals with ND highlight the heterogeneity and clinical continuum that characterizes their nature across the lifespan, which relates to different levels of cognitive ability and adaptability in behavior, affecting the person’s daily functioning and social skills [4].

Interestingly, in the *DSM-5*, all NDDs may include the specifier associated with “a known medical or genetic condition or environmental factor”. This offers clinicians the possibility of documenting specific etiological factors (e.g., genetic syndromes).

## 3. The High Rates of Comorbidity among NDDs

It is quite common for children and adolescents who received a diagnosis of a particular NDD to simultaneously experience difficulties associated with other types of NDDs. Overall, within the diagnostic grouping of NDDs, several studies provide evidence that there are high rates of comorbidity among NDDs. For example, many studies show that 22–83% of children diagnosed with ASD present symptoms that also satisfy the diagnostic criteria for ADHD [5]. In addition, ASD is often accompanied by intellectual, language, and/or motor disorders (even though they are not officially included in the diagnostic criteria of ASD).

Furthermore, several researchers stressed that the presence of emotional/behavioral (EBD) problems in this population is not solely attributed to the NDD disorder per se but rather to the fact that, very often, many of these disorders are comorbid with one another, or with other disorders (e.g., emotional, behavioral, conduct disorders) [6]. For example, many children with ADHD, at the same time, manifest autistic symptoms (and vice versa), while others experience comorbidity with ASD or SLD [7]. In addition, over 50% of children with the aforementioned disorders (ADHD, ASD, and SLD) also present symptoms of Developmental Coordination Disorder (DCD) [8]. Finally, according to [9], up to 50% of children with comorbid NDDs also present Tourette’s syndrome symptoms, often leading to temper tantrums, insomnia, obsessive–compulsive disorder, self-injuries, mood disorders, and oppositional defiant disorders or conduct disorders. All these conditions further inhibit the children’s overall functioning and emotional state [10].

The simultaneous presence of all these different types of symptoms may confuse health professionals since differential diagnosis and appropriate intervention become extremely difficult tasks. As a result, there are many cases of delayed or mistaken diagnoses [11], which, eventually, often lead to inappropriate therapeutic interventions.

## 4. Specific Learning Disorder (SLD)

Specific Learning Disorder (SLD) is a heterogeneous group of disorders with persistent difficulties in learning basic skills such as listening, speaking, writing, reading, mathematical skills, and reasoning, with normal IQ, and is considered a chronic condition that typically persists into adulthood (even cultural differences and developmental changes in how learning disabilities manifest may be evident) [4].

The *DSM-5* introduced new features in the diagnostic criteria for SLD, which are reflected in two major changes. First, SLD is categorized by ‘specifiers’ that characterize the specific manifestations of learning difficulties at the time of assessment. These include the three major academic domains, namely reading, writing, and mathematics. Secondly, there is the IQ achievement discrepancy requirement, which is replaced by four criteria (A–D), all of which must be met [1]. The recommendations for diagnosing SLD, according to the *DSM-5*, suggest that a valid diagnosis can only occur if all past and present clinical information has been selected (i.e., medical history, family and schooling background, school-based reports and observations, educational assessments, etc. [12]).

For adolescents, the presence of an SLD challenges all aspects of everyday life. These include low academic performance, low self-esteem, and a high risk for emotional and behavioral difficulties (EBD). Although most students with SLD recognize the value of hard work as a key factor in their academic success, nevertheless, their teachers often characterize them as less motivated and less competent than their peers. As a result, they rarely receive positive feedback for their efforts, which eventually leads to low self-esteem and gradual resignation [13].

According to [14], the severity of their EBDs may also be associated with the diagnosis of other (comorbid) neurodevelopmental syndromes, such as ADHD. In addition, other researchers worldwide stressed the common coexistence of conditions such as anxiety, emotional disorders, behavioral problems, and other neurodevelopmental symptoms in the presence of SLD [15,16]. The overburdened psychosocial functioning of these individuals can be evidenced either through ‘externalizing’ behaviors (e.g., impulsivity, hyperactivity, aggression, conduct problems, and/or antisocial features) or through ‘internalizing’ behaviors (e.g., withdrawal, dysphoria, and anxiety) [17]. Other symptoms such as inattentiveness, defiant behavior, conduct disorders, and a lack of communication might also be comorbid with SLD, especially during adolescence. Additional clinical externalized behaviors, such as aggression, unsociability, and misconduct, might also be evident to a level that causes deficits in their overall social and school functioning [18]. These associated features might either derive from the SLD itself and due to low learning achievement, or they might create a vicious circle of straddling deficits enhanced by the SLD (and possible comorbidity with other NDDs) presence [19].

Regarding the occurrence of SLD in adulthood despite the paucity of research, however, there is sufficient empirical evidence to suggest that SLD continues to negatively impact well-being and functioning. Furthermore, the various intraindividual characteristics; comorbidity issues; different developmental backgrounds; and varied personal, social, and occupational profiles that characterize adults with SLD underscore the enormous diversity, heterogeneity, and continuity that characterizes SLD across the life course, which creates serious difficulties both at the diagnostic and intervention levels. In a recent study [4], the following conclusion was reached: there is a need for further research and for the development of more sufficient tools for the assessment and diagnosis of SLD during adulthood, which will consider the developmental challenges and milestones in a series of domains, to assist this ‘vulnerable’ population with their life struggles.

## 5. Attention Deficit Hyperactivity Disorder (ADHD)

Since 1960, in the USA, ADHD has been identified as one of the most common NDD diagnoses in children [20], with a prevalence of approximately 3–7% among school-aged children [21]. Between 2003 and 2011, the diagnosis rate increased up to 42%, i.e., 11% (almost 6.4 million) of children aged from 6 to 17 years in the USA were diagnosed with ADHD. The onset age for ADHD was placed between 3 and 5 years of age, while almost 25% of the diagnosed children receive a formal diagnosis before the age of six [22].

Both the academic performance and the social functioning of children with ADHD are most often affected by the symptoms of inattention, hyperactivity, and impulsivity [23]. Furthermore, in almost two-thirds of the diagnosed cases, these symptoms continue to negatively affect the individuals’ life and overall functioning during the later developmental stages up to adulthood [24,25].

As regards the cognitive/learning profiles of children with ADHD, several commonly known cognitive deficiencies usually create barriers to their academic development. More specifically, research suggests that memory deficits are often evident in these students [26], along with deficiencies mainly detected in executive functions, such as a reduced ability in spatial working memory and a suspension of responses, programming, and monitoring [27]. These deficiencies are strongly related to an inability to develop efficient problem-solving skills, accomplish future goals, sustain attention and visual scanning, as well as to a reduced ‘flexibility’ [28].

It is widely accepted that cognitive abilities combined with motivation are strong predictors of high academic achievement [29]. Therefore, since children with ADHD are less proficient in time management and problem-solving skills, they tend to be more easily disorganized, less motivated, and less resistant to frustration and disappointment [30].

ADHD was characterized as one of the most ‘clinically heterogeneous disorders’, mainly due to its high rates of comorbidity with various childhood-onset disorders. For instance, 30–65% of children diagnosed with ADHD also have symptoms that are clinically significant with the diagnosis of ASD [31]. The high rates of reciprocal comorbidity between the two disorders highlight the considerable overlap of many of their symptoms.

In addition, the inattentive type of ADHD accompanies SLD in up to 70%, with the most frequent difficulties manifesting in written language production, reading, and mathematics [32]. Consequently, compared to their peers, these students have lower grades, which is often linked to their personality traits, particularly their lack of self-control [33]. This situation was linked to the issue of “school failure” and the high dropout rate in this population [34].

As it is stated [35], 40% of children with ADHD-SLD comorbidity fail to complete secondary school education. Many of their difficulties persist during adulthood since, even as postgraduate students, these individuals continue to struggle with inattention and academic issues [36].

As mentioned previously, children with ADHD and/or SLD are often faced with the danger of being stigmatized due to their ‘problematic’ academic, social, and behavioral/emotional characteristics. This is usually a consequence related to the lack of knowledge and awareness about the nature and manifestation of the related disorders, both from teachers and parents and from the wider social context. Therefore, many of these children can be easily misunderstood and characterized as ‘lazy’, ‘indifferent’, ‘aggressive’, and/or having a ‘problematic behavior’ [37], and sometimes, even ‘annoying’ in their social relationships [38].

Additional reports of comorbidity between ADHD and (S)LD range from 10 to 92% in several studies. This enormous distribution is mainly caused by the various diagnostic discrimination criteria used by different researchers [32]. For example, a recent study by De Rossi et al. [39] demonstrated a significant relationship between the clinical characteristics of youth with SLD and the inattentive type of ADHD. Other similar studies have shown that SLD was present in 70% of children diagnosed with ADHD, while 65% of these SLDs were related to written language disorders, whereas lower rates of comorbidity with ADHD were detected in the cases in which the SLDs related to reading, spelling, or math disorders [5].

Various other studies reported a high prevalence of ADHD in children and adolescents with tic disorders (e.g., Tourette’s syndrome) [40], while much higher rates of other disorders, including obsessive–compulsive disorder (OCD), oppositional defiant disorder (ODD), insomnia, mood disorders, were also found to be comorbid with ADHD [9,40].

Additional studies that investigated the co-occurrence of ADHD with internalized behavioral disorders found that depressive disorders in youth with ADHD were five times higher than in the ‘typical’ youth. The rates of comorbidity range between 12 and 50% among the several studies (e.g., [9,41]).

Many studies also documented considerable rates of comorbidity between pediatric bipolar disorder and ADHD [42,43,44]. However, empirical evidence suggests that the association between these disorders is more co-incidental than casual, as several mechanisms (i.e., shared risk factors) seem to contribute to the actual type of comorbidity [45].

The prevalence of anxiety disorders in individuals with ADHD ranges from 15% to 35% across studies [46,47]. This comorbid condition was found to negatively affect the person’s attentional issues and was associated with the development of school phobia, mood disorders, and social incompetence [9].

The most common externalizing disorders found to be comorbid with ADHD include ODD and conduct disorder (CD), as up to 30–50% of children with ADHD were also found to satisfy the diagnostic criteria for CD and ODD [48]. Comorbidity between ADHD and externalizing disorders often causes immense challenges to clinicians in terms of differential diagnosis [49].

As regards externalizing disorders and their characteristics across age (from childhood to adolescence and adulthood), a significant body of research provides evidence for a possible “shared genetic origin” [50]. In addition, there is considerable empirical evidence indicating that ADHD-like traits and early aggressive behavior symptoms seem to be exacerbated during the transition from adolescence to adulthood [51], especially if adverse family circumstances are also present [52].

Finally, children with combined ADHD and CD symptoms often also present higher rates of academic problems (e.g., SLD, reading disorders, visuospatial deficits, impaired motor or verbal skills) [52]. Comorbidity was also strongly associated with adult antisocial personality disorder, drug/substance abuse, delinquent behaviors, and/or engagement in criminal acts [53,54,55].

## 6. Discussion

### 6.1. The Possibility of a ‘Core’ Deficit Underlying NDDs

This consistent overlap of symptoms among different NDDs causes problems with the validity of specific diagnostic categories. This leads to the question of whether there is a ‘core’ deficit underlying different types of NDDs at the cognitive, neurological, and genetic levels [56].

Utilizing the discrete diagnostic categories of NDDs, several researchers conducted case–control designs to compare children diagnosed with a specific NDD (e.g., SLD–dyslexia or ADHD) with their unaffected/typical peers.

Interestingly, a core deficit was not detected in the ‘affected’ populations of the NDDs investigated in those studies. For instance, Mammarela et al. [57] conducted such a study in an attempt to identify a core cognitive deficit in Mathematical Learning Disability (MLD) (MLD is characterized as a sub-type of SLD in the *DSM-5*). Their findings indicated that neither a common (core) domain-general deficit (e.g., in working memory and in verbal or visual short-term memory) nor a domain-specific common deficit (e.g., deficits in symbolic or non-symbolic magnitude representations or poor knowledge of mathematical vocabulary) was detected in the MLD sample. Moreover, the authors claimed that similar strengths and weaknesses were found among both groups (MLD-typical children) across both domain-general and domain-specific skills. Thus, none of the possible core deficits could be described as a ‘classifier’ of MLD. The researchers concluded that children with MLD might have various deficits in both basic number-processing skills, as well as in domain-general skills; however, neither is necessarily present.

Similar findings were reported in a significant body of research studies involving students diagnosed with a specific reading disorder (or dyslexia, another subtype of SLD), i.e., with difficulty in decoding words accurately and fluently (often accompanied by spelling and/or written expression difficulties), which also attempted to identify a core deficit in dyslexia.

A common deficit that occurs in many of these studies is the ‘phonological awareness’ deficit (i.e., the individual’s ability to manipulate the sound structures of spoken words and represent them in print). However, even though poor phonological skills have long been characterized as a strong common deficit among children with reading disorders, nevertheless, not all dyslexics have poor phonological skills/awareness, and not everyone with poor phonological skills will develop dyslexia. In addition, other cognitive skills or factors were also found to play a significant role in reading difficulties. These include ‘Rapid Automised Naming’ (RAN), [58] auditory and/or language difficulties, visual-processing deficits, and memory problems [59,60].

Recently, there has been a consensus among scientists that dyslexia (SLD) is a condition that depends upon multiple risk factors operating at the cognitive, biological, and environmental levels [59,60,61]. Hence, even though there is strong evidence for the presence of a phonological deficit in the genesis of reading problems, nevertheless, no core deficit has yet been identified as ‘necessary’ or ‘sufficient’ for children to develop either dyslexia or MLD [56].

### 6.2. The SLI (CD)–SLD Overlap

Specific language impairment (SLI) and Specific Learning Disorders (SLDs) are common developmental disorders which are classified as distinct in the *DSM-5*. The term “Specific Language Impairment” (SLI) was previously used to describe children whose language development is substantially below age level and who show a considerable limitation in their overall language skills for no apparent sensory, neurological, or other deficit and despite normal non-verbal intelligence [56]. In the *DSM-5* [1], SLI was incorporated in the NDDs diagnostic grouping under the more general term “Communication Disorders” (CD).

The American Speech–Language–Hearing Association [32] and *DSM-5* [1] mention that a language disorder includes difficulties in receptive and/or expressive oral language, which usually also affects written language skills [62]. SLI prevalence ranges from 0.5% to 7% [5,63].

During the previous decades, clinicians and researchers recognized the considerable overlap between the two NDDs. For instance, it was found that approximately 55% of SLD (dyslexic) children also met the diagnostic criteria for CD (SLI), while children diagnosed with CD (SLI) also presented significant difficulties in reading acquisition [64]. Other studies provide evidence that children with CD often experience difficulties in literacy skills (reading, writing) [59,65,66,67,68] and reading comprehension [69,70,71,72]. In addition, various studies show that children with SLD often present language difficulties [64,73,74,75].

As a result, some researchers suggested that a large percentage of these children meet the diagnostic criteria for both disorders, thus confusing clinicians in terms of differential diagnosis and intervention [64]. It was even proposed that SLD (dyslexia) could be categorized as a sub-category of CD [74,76] or that SLI could be considered a more severe form of dyslexia [59]. Finally, in a particular study, it was suggested that the two disorders could be ‘incorporated’ into a single diagnostic category, which could be named ‘Language-Learning Disorder’ (LLD) [77]. It should be noted, however, that most of these studies investigated the language and learning profiles of preschool and early school-aged children, whereas adolescence is a poorly examined age period for these disorders.

Interestingly, two studies investigated the language and learning profiles of Greek adolescents who were diagnosed as either SLI or SLD using a series of diagnostic tools (e.g., intelligence; cognitive; learning skills; including reading, oral, and written language skills; and mathematics) [24,78].

One of the main findings of both studies was that the manifestation of the two disorders during childhood, adolescence, and early adulthood presents considerable differences, which might even lead the same individual to receive different diagnoses if assessed at a different age [24,78]. This might also lead to an ongoing struggle for these individuals, as they might be provided with inappropriate educational provisions or intervention programs [76].

The above acknowledgment is in line with the *DSM-5* diagnostic criteria for the SLD continuum, i.e., in how it is manifested across the lifespan. Therefore, clinicians are encouraged to seriously consider the huge changes that might occur during adolescence and adulthood in the clinical profile of the person being assessed (i.e., the alterations in patterns of strengths and weaknesses that occur across ages [13,79] and the new areas of reduced functionality that might be evident with maturation (e.g., social, interpersonal, professional) [25].

Therefore, a particular NDD may be less ‘sharp’ (distinct) during adolescence or adulthood, as many of the previous deficits or difficulties may have been resolved or improved to a certain extent, while others may have arisen [80,81,82,83].

One of the most striking findings of the study of [24] was that in the SLI (CD) group of adolescents, the ‘centrality’ of the language factor (also recognized in other studies (e.g., [76,84,85])) seems to significantly interfere with almost every basic academic domain. Even further, it seems that the language deficit even affected the overall IQ scores of this population, which in many cases was found as ‘ostensibly low’ (albeit within the normal levels). This was comprehended as a possible ‘plasmatic’ reflection of the SLI individuals’ ongoing struggle with the various academic tasks caused by their affected language skills rather than the opposite.

More specifically, both SLI and SLD adolescents seem to have overcome their initial difficulties in the areas of decoding, word-attack skills, and phonological awareness. However, the SLI group still exhibited difficulties in most of the other basic academic domains (e.g., literacy skills; oral language skills, such as vocabulary; and lack of familiarity with grammatical, morphological, and syntactical structures of written language and mathematics). By contrast, in the SLD group, written language skills (e.g., spelling and handwriting) were the only domain in which adolescents exhibited difficulties.

The clinical profiles evident in adolescents who were previously diagnosed with either SLI (CD) or SLD, i.e., the fact that the ‘typical’ characteristics of the actual disorders were not evident during adolescence, strongly question the validity of the two diagnostic categories. Similar findings were detected in the manifestation of SLD during adulthood in the studies of Bonti et al. [25,78] and in the study of [86], in which only a few “purely academic” issues were still evident in adults who were previously diagnosed with SLD in their childhood (i.e., written language skills difficulties), whereas issues in other areas of the person’s overall development and functioning were those which caused frustration to these individuals (e.g., difficulties with emotional, interpersonal, social, occupational skills).

Thus, it seems that the ongoing changes that occur in their manifestation across different ages is another issue that even further complicates the already complex nature of NDDs. Furthermore, the fact that the adolescents with SLD overcame many of the ‘basic skills’ deficiencies in language and literacy while the SLI group still struggles with almost all language and linguistic tasks indicates that the major overlap between SLI (CD) and SLD is more evident during the early school years but not in later ages.

In the study of Bonti et al. [78], the emphasis was mainly given to the clinical–cognitive profiles (particularly in mathematics) of adolescents with an SLI (CD) or an SLD diagnosis to enlighten the path for a more valid diagnosis of the two disorders during that age period. The results of this study were in line with the findings of their previous study [24], which focused on the areas of literacy (reading, writing, and linguistic skills). More specifically, the study revealed that the SLI group still encountered significant deficiencies in their overall cognitive profiles and in almost every language and mathematical measurement (e.g., concepts of numbers, executive procedural operations, word problem-solving, and mathematical reasoning). In addition, the similarity of the two groups’ profiles was mainly detected in their deficient metacognitive, metalinguistic, and meta-mnemonic strategies. In both studies, the SLD group seemed to overcome most of their difficulties (or they were less obvious) both in literacy and mathematics. On the other hand, the SLI group of adolescents still encountered difficulties with several mathematical skills, therefore confirming that impaired language skills have a strong and broad impact on the developmental continuum of mathematical ability [67].

The fact that the SLI group of adolescents still manifested weaknesses in almost all basic academic domains while the SLD group’s difficulties were evident only in very specific areas indicates that symptoms in SLD seem to reduce or become limited to specific learning domains over time. On the contrary, SLI (CD) seems to be a much more complex disorder, which, even though it strongly resembles the SLD profile during early schooling years, during adolescence, it manifests itself in the form of “generalized LD, and even leads to a ‘plasmatic’ low average intelligence level”. Hence, these considerable changes occurring with age in the manifestation of these two—initially overlapping—disorders might pose even bigger challenges in the attempts to better describe and accurately define these two NDDs [32].

In conclusion, future research should further examine the complex relationship between language and learning disorders, as well as their varied manifestation across the lifespan. SLDs can be best comprehended if viewed as multivariate dimensions where various correlated cognitive, neurological, genetic, and environmental factors contribute to an individual reaching a diagnostic threshold [61].

Furthermore, the only diagnostic boundaries for identifying a person having a disorder or not are solely quantitative (according to the ‘cut’ that divides normal from non-normal within the distribution) and do not involve any qualitative differences [87].

In addition, several factors, deficits, or impairments that lead to a particular diagnosis might also contribute to other NDD diagnoses (e.g., impaired working memory in dyslexia, MLD, ADHD). Therefore, the assignment of children and adolescents to specific categorical diagnoses is due to the clinical and practical implications. More precisely, a specific diagnosis is often crucial in terms of recognizing and successfully assisting these individuals to develop the skills that are necessary for a successful academic course and life [56].

### 6.3. The Genetics Analysis Approach

Moving away from describing NDDs solely based on behavioral phenotypes, their analysis at the genetic levels during recent years has gained much ground and popularity, especially given the rapid advances in molecular biology, genetics, and genomics [88].

As previously stated, the diagnostic categories of NDDs show considerable comorbidity and phenotypic overlap. Thus, nowadays, studies in the field of genetics try to characterize individuals with a particular NDD at the ‘etiological’ level (i.e., through the identification of recurrently observed copy number variants and/or disruptive gene variants, for example, those found in ASD (CDH8, 16p11.2, SCN2A). This led to the adoption of the so-called “genotype first” approach for diagnosing individuals with ASD [89,90].

The main task of such studies was an attempt to group NDDs based on their biological features, i.e., the specific genes and variants that were detected as causal factors. However, the enormous heterogeneity of NDDs as behavioral phenotypes is also reflected in their genetic analyses [91].

Genetic analyses were unable to come up with a single genetic diagnosis of NDDs (i.e., it was found that NDDs were not caused by one or more specific pathogenic variants detected in a single gene). Rather, many of these analyses showed that the behavioral phenotypes of NDDs were a result of various genetic events, which, in most cases, were accompanied by considerable contributions from environmental factors [92].

### 6.4. The Hypothesis of a Genetic NDD Continuum

Emerging evidence for shared genetic and environmental risk factors and predictions, which are considered as possible overlapping pathogenic mechanisms, led researchers to propose the “*Model of Neurodevelopmental Continuum*” [93]. According to this model, childhood NDDs (e.g., ID, ASD, ADHD) and adult psychiatric disorders (e.g., bipolar disorder, schizophrenia) share specific ‘genetic risk alleles’ and, therefore, are seen as representing the diverse range of outcomes caused by a disrupted or deviant brain development [93,94]. In other words, in this model, childhood NDDs and specific adult psychiatric disorders are not viewed as discrete entities, but rather, they are conceptualized and defined as lying on an etiological and neurodevelopmental continuum.

Further studies involving genetic analyses provided evidence indicating that the same pathogenic mechanisms (in the same genes or sets of functionally related genes) might be affected across disorders (e.g., ID, ASD, schizophrenia) [90,95,96].

In addition, genome-wide association studies (GWAS) also provided evidence that strengthens the hypothesis that common DNA variants contribute to the ASD phenotype, especially in cases that fall under the milder ends of the ASD spectrum [97,98].

For instance, from a genetics perspective, reading and language are both viewed as highly heritable traits, which possibly share common genetic and/or neurobiological influences [64]. Shared genetic factors were also documented between ADHD, reading disorders, and language-related abilities in several studies (e.g., [99,100,101,102,103]), while other studies found a shared genetic etiology between dyslexia and two psychiatric disorders (i.e., schizophrenia and bipolar personality disorder) [104]. Price et al.’s study [105] revealed that 22 genes that were previously associated with ASD were also detected in individuals with reading disorders.

Since the “generalist genes” hypothesis [106] was proposed, a significant body of genetics research suggests that there is an observed shared genetic susceptibility between NDDs and psychiatric disorders (e.g., [107,108]); i.e., NDDs share, at least to a certain extent, a common genetic background.

As Georgitsi et al. [91] state, studies reporting novel genomic loci and genes associated with NDDs, which show brain-specific expression (i.e., genes that are expected to be particularly important in neuronal development), might potentially assist the classification of all these disorders, and might eventually lead to better management of future treatment interventions. Examples of such genetic correlation and comorbidity among NDD phenotypes include ASD and ADHD [109,110], Tourette’s syndrome (TS) and OCD [111,112], schizophrenia and bipolar disorder [113], and, more recently, OCD and anorexia [114] or TS and ASD/ADHD [115].

Finally, neuroimaging techniques also demonstrated common neurobiological changes in NDDs, which might also be a future assessment tool in this regard [116].

### 6.5. Neurodevelopmental Disorders NDDs and Challenging/Delinquent Behaviors

Due to social and emotional difficulties, people with ND are often prone to develop challenging and/or antisocial behaviors and, in many cases, even lead to delinquency [117].

In their study, Bozas et al. [16] investigated the prevalence of clinical symptoms associated with SLD in a sample of 110 adolescents. Their findings indicated that regardless of the diagnosis (i.e., type of SLD), all individuals recorded elevated scores in the categories of clinical symptoms in tests assessing behavioral and emotional symptoms (e.g., oppositional behavior, problems in everyday life and relationships with peers, and anxiety behaviors). However, the adolescents who were diagnosed with difficulties in more than one learning area (e.g., reading, written language, and mathematics) expressed more generalized difficulties than those whose difficulties were evident in one or two academic domains. These individuals seemed to suffer from social anxiety and behavioral problems, which severely affected their relationships with their peers but also intensified their overall social functioning in their daily lives. Age did not seem to be a differential factor in terms of the symptoms detected, whereas some differences between boys and girls were detected. Overall, problems were identified mostly in all aspects of social conciliation and the social expression of anxiety, which were further negatively reinforced by their cognitive–learning deficits. The study concludes that adolescents with SLD appear to be much more vulnerable than their typical peers in almost all aspects of their psychosocial functioning [16].

As research suggests, children with NDD often show increased irritability, which was also linked to the development of future depression [118]. For instance, specific language impairment (SLI or ‘Communication Disorders’) during childhood constitutes a significant prognostic factor for future emotional/behavioral difficulties (EBD), i.e., during the adolescent and/or adult years [119].

In addition, children with ADHD present high levels of anxiety and depression and are in danger of becoming socially deprived due to their inability to follow social rules and due to their communication difficulties [120,121].

A recent study by Vish and Stolfi [122] showed that children with identified dysfunctional relationships with peers, anxiety, and/or depressive symptoms have four to eight times higher probabilities of abstaining from school for long periods. In addition, in their study, Norén Selinus et al. [8] suggest that in children with NDD, during adolescence, there is a prevalence of anti-social behavior at a level of 66, 3%, mostly among children with ASD, SLD, and DCD. Interestingly, the overall percentage of children with ADHD who claimed involvement in delinquent actions reached a rate of 41%, 31 of which (6%) were violent actions. Various other studies linked ADHD to the development of conduct problems during socialization in early life, as well as to an increased risk for anti-social activities and delinquent behaviors/actions over an entire lifespan [123].

### 6.6. Delinquent and Challenging Behaviors: Manifestation and Risk/Etiological Factors

Delinquent behavior aims at the violation of formal or informal rules. It can be expressed through several inappropriate behaviors and varies from minor (e.g., traffic violations) to severe offenses (e.g., homicide).

The following are among the most well-known etiological or risk factors that may lead to delinquency: male gender; specific personality characteristics; specific psychiatric/mental health disorders; factors occurring from the individual’s family background (e.g., individual, family, or community poverty and challenging upbringing); family dysfunction (e.g., parental criminal activity, early parental loss, parent/child separation, residential instability); adoption; abuse (physical, sexual, emotional); violence; trauma; academic/learning difficulties and/or other developmental disorders; unstable and/or disorganized neighborhoods (e.g., exposure to violence, drug-selling, crime); and substance abuse [124].

In addition, many studies specifically related delinquent behavior with several NDDs (e.g., mental retardation, specific learning and/or language disabilities, ADHD, motor coordination difficulties) or to a combination of them (comorbidity) [125]. These behaviors are often considered secondary consequences of the ongoing and prolonged periods of negative emotions and low self-esteem caused by school failure, which often lead to failure in a personal, social, occupational, and financial independence level during adulthood [24].

### 6.7. Living with NDDs over a Lifespan: Empirical Research Findings about the NDD Continuum

Other researchers identified a complex interaction of ADHD with other psychiatric or emotional/behavioral disorders (e.g., anti-social personality disorder, substance/drug abuse, conduct disorder, offending behavior, and other psychosocial dysfunctions). More specifically, the core symptoms of ADHD (i.e., inattention, impulsivity, and motor hyperactivity) most evident during childhood persist into adulthood in more than 50% of cases [126,127]. However, apart from the core symptoms, many individuals with ADHD also present problems with organizing their daily tasks and regulating their emotions, overall daily functioning, and social adaptation. Indeed, there is a significant body of research indicating that adults with ADHD (and or SLD) present serious impairments in their interpersonal relations, both with family members and peers. At the same time, an increased rate of separations and divorces was recorded among the adult ADHD/SLD population [123].

Additional studies also reported higher rates of traffic violations and accidents among the ADHD adult population, violations of rules and delinquent behaviors, as well as an increased prevalence rate of an ADHD diagnosis among juvenile and adult offenders [128]. Furthermore, a meta-analysis also validated the strong relationship detected between ADHD/SLD and delinquent behaviors [129], whilst numerous longitudinal studies also revealed a high correlation between the prevalence of an ADHD/SLD diagnosis and incarcerations [130]. Significant associations between childhood ADHD and adolescent/adulthood arrests related to drugs and violence were reported in a study [131]. High percentages of adulthood ADHD diagnosis were also reported in studies that investigated in-prison and within-prison settings, with no significant gender differences reported (e.g., [132]).

Types of criminal activities, such as assault, theft, drug-related crimes, and possession of weapons, were the most frequent reasons for contact with the justice system among ADHD-diagnosed offenders [133]. Finally, an association of hypersexuality and ADHD symptomatology was reported in a recent study [134], whereas other studies report a 35.8% prevalence of an ADHD diagnosis in their sample of 120 paraphilic and hypersexual men, two conditions highly considered as risk factors for sexual offending [135].

Further evidence of the complex interplay of causation among SLD adults’ clinical profiles, socio-demographic characteristics, and emotional and behavioral issues is provided in the study of Bonti et al. [24]. More specifically, the study revealed that in a sample of 350 Greek adults (226 male–124 female) who requested a diagnosis for possible SLD between 2012 and 2018, 73% were diagnosed with SLD (or SLD with comorbid ADHD or other NDD at 54%), 68.5% had normal intelligence, 70.4% completed their Secondary Education, 26.1% completed their Postsecondary Education, and 3.5% only completed their Primary Education. The main reason for their referral request was participation in several types of academic exams (77%). This finding was interpreted since adults in Greece seek learning assessment due to socio-educational reasons and reasons with a socio-economic orientation. These reasons also relate to later academic development and lead to better vocational rehabilitation. Finally, 80.0% of the participants were single, 14.6% were private employees, 6.0% were public or local employees, 5.0% were self-employed, 20.6% were unemployed, 43.5% were students, and 10.3% were employed students.

The overall analysis of the demographic characteristics of adults with SLD led to the following revelation: most of the sample were young (male) adults (up to 30 years), Secondary Education graduates, still single, still studying, unemployed, financially dependent, and with poor social and personal lives.

According to the *DSM-5* diagnostic criteria, to receive a diagnosis of ADHD during adulthood, apart from the persistence of core symptoms, clear evidence is required that symptoms interfere with the individual’s functioning across various domains of everyday life [123]. According to the findings of the above study, the complex nature of SLD agrees with the *DSM-5*’s new diagnostic criteria (i.e., the suggestion of a multi-factorial assessment and analysis). More precisely, the findings of the study point out the ongoing struggle of SLD adults to obtain academic qualifications and to gain vocational rehabilitation, along with their difficulty in creating a family, possibly because of their lack of occupational status, their financial insecurity, and the emotional/self-esteem issues they usually encounter due to their ongoing learning problems.

Hence, SLD has a developmental nature and continues to influence several domains of a person’s life (even during adulthood). In addition, the high level of comorbidity (54%) interferes with the existing problem of establishing a common clinical profile for adults with SLD [4,25].

In conclusion, it is of great interest that most of the above characteristics of youths and adults with SLD are correlated with the risk factors for delinquency in the general population, which logically leads to the following question: does having an SLD (or NDD) lead to delinquency [24]?

### 6.8. Theoretical Approaches Linking SLD (and Other NDDs) with Delinquency

Various contemporary theoretical approaches attempted to identify the possible reasons why adolescents with learning disabilities may often exhibit delinquent behavior and issues with justice [136,137,138,139]. Four of the most dominant of these theoretical approaches are as follows: (a) The School Failure Hypothesis, according to which learning disabilities lead to poor academic performance, and consequently, this leads to the negative treatment of young people by parents, teachers, and peers. This situation raises in young people the need to socialize with like-minded others who have similar experiences. Also, academic “failure” can trigger a tendency toward truancy, school problems, and delinquency [140]. (b) The Susceptibility Theory. According to this theory, learning disabilities often create physical and personal problems that make the person vulnerable, predisposed, and/or prone to delinquency, such as disruptive behaviors in organized settings and rule violations [141]. (c) The Differential Treatment Theory supports that children with learning disabilities have an equal chance of engaging in delinquency as their typical peers and are more likely to be treated differently by their peers and adults. This is the phenomenon of being targeted by the social environment (school, juvenile justice, society) and results in an overrepresentation of youth with LD in juvenile correctional institutions [142]. (d) Moffitt’s Theory emphasizes mostly the biological factors rather than the social ones. While most theories focus on how young people with learning disabilities interact with social contexts, Moffitt’s theory [143] places greater emphasis on the neurological deficits associated with disabilities and delinquency [144].

More specifically, Moffitt distinguished two types of offenders: (a) the “adolescent-limited type” and (b) the “life-course persistent type”. The first refers to young people with antisocial behavior, even though their childhood was not recorded as problematic. Delinquent behavior in this type starts at the beginning of adolescence, and in adulthood, this behavior usually disappears. In the second type, neurological deficits (e.g., LD or other NDDs) are considered the main contributor to their delinquent behavior throughout their life course. Opposite to the first type, these individuals engage in aggressive or delinquent behaviors at preschool age (3–4 years), while as teenagers, they show frequent and severe antisocial behavior, and the delinquent behaviors are more likely to continue into adulthood [143,144].

The common factor to all the above theoretical perspectives is a similar conceptualization, i.e., having a learning disability, which may somehow contribute to aggressive, challenging, and/or delinquent behavior. Therefore, undoubtedly, an individual is placed in a ‘youth at risk’ population if diagnosed with NDD [145]. The presence of an SLD or other NDDs often makes the person socially and emotionally vulnerable, a condition often referred to as a ‘traumatized youth’ [146]. This emotional vulnerability or ‘personal trauma’, caused by commonly accepted risk factors (such as NDDs), increases the likelihood of involvement in delinquency or other antisocial and/or problem behaviors [147].

Furthermore, the literature focusing on the co-existence of ADHD-related problems and delinquency is more extended than the research focusing on the relationship between SLD and delinquent behavior [129,148]. Studies highlighted the increased rates of delinquency among adolescents diagnosed with ADHD, both in terms of minor delinquency and crimes resulting in institutional confinement [149]. Previous research showed that compared to their counterparts without LD, adults with LD had significantly higher incidences of violent acts and substance abuse, such as tobacco and drug use (such as marijuana), according to self-reports and several survey data [150], given that most research focused separately on youth diagnosed with LD or ADHD and not on comorbid cases (e.g., [124,150,151,152]). Some of the few studies that examined comorbid ADHD and LD and/or other disorders cases revealed that these adolescents used more nicotine and marijuana, along with more frequent participation in acts of direct aggression and/or minor delinquency [129,153].

As already mentioned, comorbidity between ADHD and SLD is present in a large percentage of children diagnosed with NDD. Therefore, the causal link between SLD and delinquency is formed through a more complex network when the factor of comorbidity is added.

Finally, there is a strong possibility that both ADHD and SLD are factors that also correlate with many additional or secondary disadvantages (e.g., gender, race, socioeconomic status) [124]. To this end, it could be argued that neither SLD nor ADHD itself is directly causally related to delinquency, but the propensity for delinquent behavior may also be triggered by other risk factors commonly recognized in the literature as being highly correlated with the prevalence of an NDD (e.g., low self-esteem, anxiety, depression, poor social skills) due to the increased social stigma associated with having an SLD or other NDD, which may result in aggressive or delinquent behaviors as reaction mechanisms [129] and not cause this kind of behavior.

### 6.9. The Concepts of Giftedness and Twice Exceptionality

Many definitions of “giftedness” were recorded in the relevant literature, which argues that “gifted” students are characterized by high intelligence or some specific talent (or both), either in a specific domain or across a whole range of domains [154]. In previous years, giftedness was diagnosed by a high level of intelligence (Spearman’s ‘g’ factor of general intelligence) [155]. More recent definitions of giftedness incorporated various intelligence or personality characteristics referred to as ‘multi-dimensional’ models, such as Gardner’s [156] multiple intelligences model, Renzuli’s [157,158] ‘*Three Ring Model*’ of high ability (which described the gifted achievement as a combination of high general intelligence, task commitment, and creativity) and Gagne’s [159] differentiated model of giftedness and talent [160,161]. They were also included in more recent conceptualizations of giftedness: (1) ‘superior intelligence level’, (2) ‘academic superiority’, (3) ‘leadership skills’, (4) ‘creativity’, and (5) ’artistic skills’ [162].

On the other hand, there is a distinction between gifted and talented students, with the former described [163] as students with exceptional natural abilities in one or more areas of human ability (e.g., intellectual, creative, social, or physical abilities) and the latter described as students capable of transforming their “giftedness” into exceptional performance, which may also demonstrate high levels of ability in specific areas of human ability.

However, there is consensus among researchers that gifted and/or talented (g/t) students are a highly heterogeneous population, exhibiting high intellectual abilities and/or talents in various domains (e.g., cognitive, creative, artistic) but may also display varied and different interpersonal characteristics [154].

Although gifted or talented students are expected to have exceptional potential, they may nevertheless often underperform or fail in some areas. Thus, on the occasion of a group of learners with learning difficulties and academic strengths or talents, the term was introduced to “twice exceptional” students who have coexisting diagnoses of both Special Educational Needs and who have been officially recognized as gifted/talented. As with the concept of giftedness, the double exception condition also puzzled researchers as to how it should be defined [164,165]. One of the definitions that received substantial consensus among twenty-six organizations supporting the research and educational needs of this population was proposed by Baldwin et al. [166]. According to this, ‘Twice-exceptional individuals’ evidence exceptional ability and disability, which results in a unique set of circumstances. Their exceptional ability may dominate, hiding their disability; their disability may dominate, hiding their exceptional ability; each may mask the other so neither is recognized or addressed’ (p. 212).

The most seen overlap and confluence in the US is that of Specific Learning Disabilities (SLD), which focuses primarily on the significant discrepancy between a student’s level of ability and their academic performance [167]. However, more recent studies revealed that these co-occurrences of gifts/talents and disorders are not limited to a specific neurodevelopmental–intellectual disorder (e.g., ASD and SLD) but are phenomena recorded across the broader spectrum of developmental disabilities [165,168].

Regarding the prevalence of twice-exceptional students, few data were recorded internationally. However, it is now clear that such students exist quite often in various educational contexts [167]. Twice-exceptional students are considered to be at particularly high risk for educational failure and poor outcomes due to insufficient recognition and support [169]. Consequently, as with NDDs, there is an increased likelihood of high co-occurrence of students with these characteristics as well as EBD, which may also lead to aggression, problems, or delinquent behaviors [165]. However, more research is still needed on the etiology of twice-exceptionality, let alone its co-occurrence with EBD-related diagnoses [165].

## 7. Future Directions

### 7.1. Implications for Diagnosis and Intervention

High rates of comorbidity between NDDs recently caused concerns regarding the considerable possibility that NDDs (or at least NDDs in particular), in many cases, either share a common genetic etiology [170,171,172] or have a distinct genetic origin, (which causes their co-occurrence) [173] or they are influenced by the same genes, as in the case of ADHD and SLD (dyslexia).

Other studies documenting comorbidity among ASD (autistic) traits, motor coordination problems, reading disorders (SLD), and executive functioning deficits also suggested the possible prevalence of a shared underlying neuropsychological dysfunction [47,174]. As Ter-Stepanian et al. [175] argue, the co-occurrence of NDDs could be partly due to shared familial/heritable or neuropsychological deficits and motor dysfunctions. All these hypotheses cause serious doubts concerning the conceptualization of NDDs as distinct phenotypic entities [175,176,177].

All the above conceptualizations also have implications regarding successfully treating these conditions, i.e., finding better ways of successfully meeting the needs of these individuals, especially within the school context. If this is the case, then treating each of these disorders independently would be an unsuccessful approach [178]. Examples of risk for mistreatment may be derived from studies that investigated the response of individuals with different comorbidities to several types of treatments/intervention procedures. For example, even though behavioral therapy helped treat individuals with comorbid ADHD–anxiety disorders, nevertheless, people with comorbid ADHD and ODD or CD were much less responsive to single behavioral therapy. Rather, these individuals responded significantly better when behavioral therapy was combined with medication and/or other psychotherapeutic interventions [47,176].

Hence, a better and more comprehensive description of ‘comorbidity profiles’ that would be based on cross-disciplinary/inter-disciplinary diagnostic approaches, which would combine genetic, phenotypic descriptors, core deficits, and treatment responses [179], might lead to a more individualized and appropriate diagnostic and intervention system.

In addition, educational systems should adapt their teaching methods based on the learners’ strengths and weaknesses and, therefore, view these disorders within a multi-dimensional approach rather than based upon a single diagnosis.

### 7.2. Future Research

The same implications apply to future research studies, which should focus on capturing the heterogeneity in NDDs and should consider the whole distribution of multiple correlated factors instead of conducting traditional case–control research designs that take as a prerequisite a specific diagnosis (e.g., ADHD, SLD, ASD) [180,181].

Future research should aim at the development of a more comprehensive ‘screening’ for comorbidity cases in the NDD population to better meet their individual needs in all life domains and provide them with a better ‘quality of life’.

Additionally, this paper aims to introduce the above conceptualization and address these considerations regarding the diagnosis of different types of NDDs. However, from a specific scientific perspective, that of psychology and special education, it is recommended that it would be appropriate to ‘dig deeper’ in areas broader than these scientific fields.

The prerequisites for such an innovative change, however, are multi-dimensional. They involve, among others, the development of a new diagnostic system (possibly with comorbid conditions), mandatory comprehensive screenings in all educational contexts (as early as possible), changes in legislation, and professional and teacher-training programs.

## 8. Conclusions

The aim of the current article was not to review all the empirical findings of the genetically based diagnostic procedures, the neurobiological and neuropsychological fields, and the fields of EEG and proteomics (related to the diagnosis of NDDs). Rather, it aimed to address the issue of high rates of comorbidity among different types of NDDs regarding the difficulties caused (due to these high rates of comorbidity) in the overall diagnostic procedure, which is currently based on the distinct diagnoses (e.g., ASD, SLD, ADHD) of the *DSM-5*.

The new trend of placing NDDs within a spectrum, rather than classifying them as discrete entities, that recently arose is due to the increasing appreciation of the significant levels of phenotypic overlap between NDDs. This spectrum is evidenced in the *DSM-5*, where all previous subtypes of, for example, SLD or ASD (e.g., specific reading disorder and Asperger’s syndrome) were subsumed to a single diagnostic label. Rates of comorbidity between reading disorder (dyslexia) and other neurodevelopmental disorders vary widely, but, on average, about 40% of children with a reading disorder will also have another disorder, highlighting that the need for extending and specifying existing causal models is more necessary than ever [182,183].

Maybe the term Neurodevelopmental Spectrum Disorders (NSD) should be introduced, which might include, apart from the already separate types of NDDs, EBDs, and twice-exceptional learners and the new diagnostic criteria, clinical characteristics from all the above disorders. This way, a multi-professional diagnostic group would be encouraged to map out the learners’ overall cognitive, behavioral, and learning (neurological or genetic) individual profiles. As ASD or SLD incorporated the previously known subcategories, maybe NSD should incorporate all possible combinations of disorders or comorbid characteristics from various disorders.

The continuum of NDDs is also very interesting, and it leads us to think that the NDD spectrum idea might be the answer to the high comorbidity levels of NDDs, as it seems that almost all NDDs lead to the same picture during the adult years (every aspect of the individual’s life is finally affected). Does this get us back to previous theories (e.g., Moffit’s theory) [148]? There is a core ‘neurological’ (or maybe genetic) disorder that is bound to lead the person to similar life issues, irrespective of their clinical characteristics during the early years; i.e., is it expressed “in the form” of ASD, ADHD, SLD, or a combination of them?

The NDD continuum hypothesis, along with the dramatic acceleration of genome and whole-exome sequencing analyses during the past decade [88], has considerable implications for both diagnostic procedures and intervention planning. More precisely, these innovative conceptualizations call for the development of a new, more comprehensive, and flexible diagnostic system for NDDs and other mental disorders. In addition, the benefits of these models might be substantial in therapeutic, individualized intervention planning, both at a clinical and psychosocial level, for better responding to the needs of individuals who present symptoms of NDDs or psychiatric or other disorders, albeit based on a more “open-minded” diagnostic framework.

In the quest to unravel the complex architecture of the NDD phenotypes, several methodological approaches were utilized throughout the last decade. These include genetic sequencing studies, long-scale high-throughput genome-wide genotyping studies, various novel gene association studies, and other biological processes, as well as studies examining possible alterations in brain anatomy, connectivity, and function (assessed via neuroimaging techniques). In future research, the progress of these innovative methods and other fourth-generation sequencing and neuroimaging methods will hopefully manage to fill in the knowledge gaps between genomics, molecular pathways, cellular communication, neuronal cognition, and brain function. The ultimate goal is to manage to translate these valuable measures, as well as better assessment and intervention techniques and tools [91,184,185] to improve the diagnostic approaches to neurodevelopmental disorders.

## Data Availability

No new data were created or analyzed in this study. Data sharing is not applicable to this article.
